# Nurses’ opinions on euthanasia in Spain: an evaluation using a new version of the EAS

**DOI:** 10.1186/s12912-024-02176-5

**Published:** 2024-07-29

**Authors:** Daniel Lerma-García, María Laura Parra-Fernández, Cristina Romero-Blanco, María Soledad Olmo-Mora, María Dolores Onieva-Zafra

**Affiliations:** 1 Department of Nursing, University of Illes Balears. Ibiza, Baleares, Spain; 2 Faculty of Nursing of Ciudad Real, University of Castilla-La-Mancha, Camilo José Cela 14, Ciudad Real, 13071 Spain; 3 Bienestar Social de La Junta de Comunidades de Castilla-La Mancha, Ciudad Real, Spain

**Keywords:** Euthanasia, Attitude, Instrumental validation, Psychometric properties, EAS

## Abstract

**Background:**

Bioethical dilemmas at the end of life have led to regularization processes for the provision of medical assistance in dying patients in different countries. Since the regulation of euthanasia in Spain in 2021, the euthanasia act has been included as one of the benefits of the health system, which has undergone uneven development and implementation in different autonomous communities. The aim of this study was to review the Spanish version of the Euthanasia Attitude Scale following the partial modification of four items.

**Methods:**

A cross-sectional study was conducted with a non-probabilistic sample of Spanish health workers from Islas Baleares. A self-reported sociodemographic questionnaire and the Euthanasia Attitude Scale were used for data collection. The psychometric properties of the scale were assessed, including reliability and validity, using a confirmatory factor analysis and a parallel analysis.

**Results:**

The Cronbach's alpha of the EAS was α = 0.892, which implies good internal consistency. According to the confirmatory factor analysis, a Kaiser–Meyer–Olkin–value of 0.938 was obtained, and the result of Bartlett's test of sphericity was < 0.001. The questionnaire included four loading factors, which explained up to 56.99% of the variance. The parallel analysis revealed three significant factors and a fourth, less interpretative factor.

**Conclusions:**

The EAS-ES-R is a valid instrument for assessing the attitudes toward euthanasia of both trainees and practicing health professionals. It may also be of vital importance in detecting training, support and implementation needs for laws regulating euthanasia in Spain.

## Background

In the bioethical field, the word euthanasia refers, based on its etymology, to “good death” [1]. In reference to the conceptual development of ethical conflicts related to the end of life, different terms have been used to focus on the bioethical debate and its implications for health care [2]. There is current bioethical consensus on the contextualization of these conflicts in two scenarios: euthanasia and assisted suicide [3, 4]. These two concepts are integrated within "medical assistance in dying" (MAID), which has been regulated in different countries [5–8].

In the Spanish context, this regularization took place because of Organic Law 3/2021 on the regularization of euthanasia, which seeks to provide a legal response to social demands regarding ethical conflicts related to the end of life [9]. The text includes the concept of MAID, which refers to “the set of services and assistance that healthcare personnel must provide, within the scope of their competence, to patients who request the necessary aid in dying”. It also states that these benefits must be provided “in accordance with certain conditions that affect the physical situation of the person with the resulting physical or mental suffering, the possibilities of intervention to alleviate their suffering, and the moral convictions of the person regarding the preservation of their life in conditions that the person considers incompatible with their personal dignity”. In this sense, guarantees must be established to ensure that the decision to end one’s life is made with absolute freedom, autonomy and knowledge, thus protected from pressures of any kind that could come from unfavorable social, economic or family environments, or even from hasty decisions". In this context, nurses have been included in the healthcare team where in collaboration with the doctor, they will provide the necessary assistance to the patient until the moment of death. Thus, in the context of the Balearic Islands [10, 11], new roles have been introduced within nursing practice, such as for drug administration nurses, who possess competence profiles aligned with intensive care and emergencies. Furthermore, there are nurses specializing in end-of-life care, providing support in both primary and specialized settings and offering guidance to fellow professionals. The work performed by these professionals spans from the moment a euthanasia request is made to postmortem care [12].

The implementation of this law in the Spanish territory has been uneven due to the controversy it has sparked and the varying ethical and anthropological sensitivities among the different regional governments in Spain. Despite its legalization, the debate persists within the various political and social spheres of our sociocultural context. In certain regions, its enforcement has faced obstacles, as health professionals have the acknowledged right to exercise conscientious objection. According to Spanish law, this grants health professionals the individual right to refrain from participating in health actions regulated by this law if they are incompatible with their own convictions [13–16]. Research on the attitudes of health professionals toward euthanasia using various measurement instruments has been conducted in different countries with varying regulations regarding its application, thus reflecting diverse sociocultural contexts [17–29]. In this sense, understanding the attitudes of health professionals after the application of the law in recent years is crucial for promoting strategies that ensure its full implementation and establishing scenarios that guarantee the rights of both citizens and health professionals.

The Euthanasia Attitudes Scale (EAS) is an instrument designed to assess respondents’ attitudes toward euthanasia. Originally proposed and validated by Tordella and Neutens [30] in 1979 in the United States, it was later modified by Rogers [31] in 1996. Subsequently, the scale underwent further modifications to assess social values and ethical judgments regarding euthanasia [32]. The Euthanasia Attitudes Scale (EAS) has additionally been adapted and validated in various languages (refer to Table 1), demonstrating its applicability across different sociohealth contexts, albeit with variations in its structure [33–36]. The initial validation of this instrument in Spanish occurred during the discussion phase of the law when euthanasia was not legal in Spain. Following this preliminary exploratory analysis and a few years after the law's enactment, it became necessary to review the questionnaire's structure and its individual items. The objective of the current study was to conduct a new validation of the EAS instrument, incorporating confirmatory analysis and readapting some of its items.

**Table 1:** Summary of the characteristics of the study and its validation methodology prior to the EAS-ES-R

**Ref**	**Language**	**Sample type**	**Sample size**		**Validity reported (Cronhbach)**	**Method: CFA, EFA**	**Factor Structure**	**items**	**Fit Indices**
**Tordella et al. (1979)** [30]	**English**	**Nursing students**	**N = 150**	**EAS**		**Test–retest** **Correlación de Pearson**		**21**	
Rogers [31] [31]	**English**	**Nursing students**	**N = 216**	**EAS-R**	**0,84**	**CFA**	**3**	**18**	**-CFI = 0,92** **-RMSAl = 0.82**
**Onieva-Zafra et al. (2019)** [32]	**Spanish**	**Nursing students**	**N = 396**	**EAS-ES**	**0,878**	**EFA**	**4**	**21**	**-KMO = 0,905** **- Bartlett's test of Sphericity = 2972.79 (*p* < 0,001)**
**Malliarou et al. (2022)** [33]	**Greek**	**physicians**	**N = 93**	**Gr-EAS**	**0,944**	**CFA**	**5**	**30**	**-KMO = 0,868** **-CFI = 0.953** **RMSA = 0.08**
**Chong et al. (2004)** [34]	**Chinese**	**Social workers students**	**N = 618**	**EAS-EC**	**0,77**	**CFA**	**4**	**31**	**-X2 = 1525,24** **-CFI = 0,96** **-RMSA = 0.54**
**Demedts et al. (2023)** [35]	**Dutch**	**Nursing students**	**N = 273**	**EAS-UMS-NL**	**0,822**	**CFA**	**4**	**21**	**-KMO = 0,898** **- Bartlett's test of Sphericity = 2180.787 (*p* < 0,001)**
**Aghababaei** [36]	**Arabic**	**Nursing students**	**N = 233**		**0,88**	**EFA**	**3**	**20**	**-KMO = 0,93** **- Bartlett's test of Sphericity = 90,3 (p = 0,0001)**

### Aim

The study’s specific objectives were to readapt and validate the EAS Scale for use in a Spanish context, to assess the scale's dimensionality through confirmatory factor analysis (CFA), and to determine the scale's reliability.

## Methodology

### Sample

All health professionals of the Balearic Health Service, nursing degree students and specialist professionals in training in the Balearic community were invited to participate in the study. For the recruitment of the sample, once authorized by the different management practices through the respective research commissions, collaboration was requested for the dissemination of the questionnaire by global mailing to all professionals. To calculate the sample size, we took into account the criterion of performing the confirmatory analysis, whose criteria included a minimum of 10 subjects per test item. In this case, our sample should be a minimum of 210 subjects with a loss range of 60 subjects.

The data were collected over six months, between July and November 2023, for a total of 828 answers. The questionnaire was completed anonymously and confidentially. The inclusion criteria included being a health professional contracted in any of the Balearic health system management offices, being a nursing degree student or being a professional in training as a specialist in the Balearic community, understanding the language and concepts used in the instrument, agreeing to participate in the study and completing the informed consent form authorizing the use of the information for scientific purposes.

### Procedure

Ethical approval for this study was obtained from the Research Ethics Committee of the Balearic Islands Ethics, according to the ethical guidelines established by the Helsinki Declaration in 2008 (Code number CEI: IB 5116/23 PI). The study included a patient information sheet about the project and an informed consent form. All participants were informed about the project and provided informed consent by completing and submitting all the questionnaires. The data obtained are confidential and cannot be used for any purpose other than the objective of this study.

This study was conducted in two steps: (1) revision of the EAS-ES scale items through a new cross-cultural translation and adaptation of the EAS into Spanish and (2) a psychometric assessment of the validity and reliability of the new version obtained (EAS-ES-R). The development of the preliminary version of the scale followed the usual recommendations (30).

Two translations of the original version into the language of the target population were retranslated by bilingual translators whose mother tongue was Spanish. Subsequently, a back-translation was carried out by professionals whose mother tongue was English. Subsequently, the research team reviewed the previously validated EAS-ES version and the newly translated version. A second team, composed of members of the Health Care Ethics Committee of the Ibiza and Formentera Health Area, reviewed it to obtain semantic and technical equivalence in appropriate bioethical language. Four items were modified with respect to the previously validated translation into Spanish. A pilot study of the final draft was carried out with 10 Spanish-speaking participants and healthcare professionals to assess the comprehension and suitability of the questionnaire and to review the fluency, readability and comprehensibility of the items. The professionals reported no major difficulties. This resulted in the final version of the EAS-ES-R questionnaire. After translation to Spanish and back-translation to English, a revision made by the principal investigators resulted in 4 modified items for the new version of the EAS-ES-R scale (Table 2).

**Table 2:** Revision of items for the EAS-ES-R

Ítem EAS	New ítem EAS-ES-R
2. Inducing death for merciful reason is wrong	2.Inducir la muerte por compasión es incorrecto
4. There are never cases when euthanasia is appropriate	4.No existen casos en que la eutanasia sea apropiada
7. Euthanasia should be against the law	7.La eutanasia debe ser ilegal
15. I have faith in the local medical system to implement euthanasia properly	15.Confío en el sistema de salud para implementar la eutanasia adecuadamente

### Instruments

The Euthanasia Attitude Scale (EAS), developed by Tordella and Neutens in 1979 [30], consists of 21 items organized into four domains: ethical considerations (11 items), practical considerations (four items), treasuring life (four items), and naturalistic beliefs (two items). The respondents rated their agreement with each item on a five-point Likert scale: 5 = strongly agree, 4 = agree, 3 = neither agree nor disagree, 2 = disagree, and 1 = strongly disagree. The total score ranged from 21 to 105, with higher scores indicating more positive attitudes toward euthanasia. Example items include "A person with a terminal illness has the right to decide to die" (ethical considerations), "Euthanasia is acceptable if the person is old" (practical consideration), "There are very few cases when euthanasia is acceptable" (treasuring life), and "A person should not be kept alive by machines" (naturalistic beliefs). The scores of several items are reversed: 1b, 1d, 1 g, and 1i for the first original factor; 2c for the second factor; 3a and 3c for the third domain; and 4b for the fourth. Additionally, items 3b and 3d were reversed. The original internal consistency index was reported as 0.84 [31].

Demographic variables, such as sex, age, marital status, years of experience, knowledge of ethics, knowledge of the new euthanasia law, and religiosity, were included in the sociodemographic questionnaire.

### Analysis

The statistical analyses were performed using IBM SPSS AMOS version 26.0. First, the data were coded and explored, after which new variables were calculated taking into account the correction criteria of the questionnaire used. Second, a descriptive analysis of the set of variables was carried out to determine the composition of the sample; frequencies with percentages for categorical variables and the means and standard deviations for quantitative variables were used for the descriptive analysis. Third, the Kolmogorov‒Smirnov test (N > 50) was applied to determine whether the quantitative variables conformed to the normal curve. Reliability was studied through Cronbach's α. In addition, a confirmatory factor analysis and a parallel analysis were carried out. In all cases, we worked with a confidence level of 95%. For the CFA, several indices were applied to determine the overall fit of the factorial solution: X2, adjusted goodness-of-fit index (AGFI): values over 0.90 imply an optimal model; comparative fit index (CFI), normed fit index (NFI) and Tucker‒Lewis coefficient (TLI). In all cases, the range of values should be between 0 and 1, and the reference value is 0.90 [19]. Thus, the standardized random mean square residual (SRMR) and root mean square error of approximation (RSMEA) were also checked for overall fit. In this sense, for both indices, lower values involve better fit, with a reference value of 0.08 [20].

## Results

### Sociodemographic variables

The sample consisted of 828 participants, 76.1% of whom were women and 23.8% of whom were men, with ages ranging from 18 to 72 years (M = 42.28; SD = 11.34). With regard to the country of birth, 92.1% were born in Spain, 2.2% were from other EU countries, and 5.7% were from non-EU countries. Regarding marital status, 45.2% declared themselves single, 44.9% were married, 8.8% were separated/divorced and 1.1% were widowed and a total of 51.6% had children. In terms of religion, 74,9% considered themselves religious. With respect to the length of professional practice, 31,5% had been working between 5 and 10 years, 28.7% between 11 and 20 years, 26.5% between 21 and 30 years and 13.3% for more than 30 years. Regarding the knowledge of Law 3/2021 on the regulation of euthanasia, 6,5% did not know anything at all, 61,8% acknowledged that they knew little about it, and 29% knew a great deal or completely about it. Forty-nine percent of those surveyed stated that they had attended some training on ethics, with 49% of those surveyed considering themselves sufficiently trained in this respect. A total of 69.5% of professionals acknowledged that they had never attended any training on euthanasia (see Table 3).

**Table 3:** Sociodemographic results

	**f**	**%**
Gender		
Male	197	23,8
Female	630	76,1
Age		
18–27	97	11,7
28–37	196	23,7
38–47	237	28,7
48–57	211	25,5
Más de 58	84	10,1
Religiosity		
yes	208	25,1
No	620	74,9
Marital Status		
Soltero/a	374	45,2
Casado/a	372	44,9
Separado-divorciado/a	73	8,8
Viudo/a	9	1,1
Occupation		
Nurse	360	43,5
Physician	167	20,2
Pharmacist	7	0,8
Physioterapist	14	1,7
Social Worker	14	1,7
Psicólogist	2	0,2
Other healths professionals	44	5,2
Profesional specialization		
yes	273	55,1
No	335	44,9
Years of profesional experience		
0–5 years	121	16,2
6–10 years	114	15,3
11–15 years	102	13,7
16–20 years	112	15,0
21–30 years	198	26,5
More tan 30 years	99	13,3
Family member with incurable illness		
yes	657	79,3
No	171	20,7
Knowledge of euthanasia Law		
None	52	6,5
Little	512	61,8
Much	189	22,8
Completely	51	6,2
Involvement in patient care within a euthanasia context		
yes	130	15,7
No	674	81,4

### Ethical variables relating to euthanasia

Regarding the knowledge of Law 3/2021 on the regulation of euthanasia, 68.3% of the respondents recognized that they knew little or nothing about it, and 29% knew a great deal or completely about it. Forty-nine percent of those surveyed stated that they had attended some training on ethics, with 49% of those surveyed considering themselves sufficiently trained in this respect. A total of 69.5% of professionals acknowledged that they had never attended any training on euthanasia.

### Reliability analysis

The Cronbach's α for the EAS is 0.892, indicating good internal consistency (see Table 4).

**Table 4:** Means, standard deviations, item homogeneity, and α if the item is deleted from the Euthanasia Attitude Scale (EAS)

	**M**	**SD**	**Item-total correlation**	**Cronbach’s alpha if item deleted**	**Asymmetry**	**Kurtosis**
1. 1a. A person with a terminal illness has the right to decide to die	4,47	0,902	0,536	0,886	-2,309	5,685
2. 1b. Inducing death for merciful reason is wrong	3,13	1,224	0,348	0,892	-0,178	-0,929
3. 1c. Euthanasia should be accepted in today’s society	4,40	1,224	0,348	0,892	-1,887	3,954
4. 1d. There are never cases when euthanasia is appropriate	4,16	0,993	0,565	0,885	-1,342	1,654
5.1e. Euthanasia is helpful at the right time and place	4,36	0,871	0,666	0,883	-1,843	3,983
6. 1f. Euthanasia is a human act	4,27	0,928	0,727	0,881	-1,528	2,374
7. 1 g. Euthanasia should be against the law	4,52	0,854	0,673	0,883	-2,410	6,408
8. 1 h. Euthanasia should be used when the person has a terminal illness	3,43	1,134	0,405	0,890	-0,304	-0,826
9. 1i. The taking of human life is wrong no matter what the circumstances	4,07	1,081	0,642	0,883	-1,396	1,491
10. 1j. Euthanasia is acceptable in cases when all hope of recovery is gone	3,89	1,013	0,602	0,884	-0,985	0,541
11. 1 k. Euthanasia gives a person a chance to die with dignity	4,43	0,879	0,751	0,881	-1,964	4,118
12. 2a. Euthanasia is acceptable if the person is old	2,60	1,127	0,291	0,893	-0,534	-0,496
13. 2b. If a terminally ill or injured person is increasing concerned about the burden that his/her deterioration of health has placed on his/her family, I will support his/her request for euthanasia	3,37	1,115	0,497	0,887	-0,307	-0,692
14. 2c. Euthanasia will lead to abuses	3,69	1,028	0,566	0,885	-0,524	-0,216
15. 2d. I have faith in the local medical system to implement euthanasia properly	4,05	0,967	0,619	0,884	-1261	1,603
16. 3a. There are very few cases when euthanasia is acceptable	3,54	1,090	0,443	0,889	-0,465	-0,583
17. 3b. Euthanasia should be practiced only to eliminate physical pain and not emotional pain	3,90	0,965	0,225	0,894	-0,921	0,819
18. 3c. One’s job is to sustain and preserve life, not to end it	3,87	1,114	0,667	0,882	-1,141	0,649
19. 3d. One of the key professional ethics of physicians is to prolong lives, not to end lives	3,86	1,050	0,567	0,885	-1,069	0,689
20. 4a. A person should not be kept alive by machines	3,27	1,087	0,039	0,900	-0,061	-0,833
21. 4b. Natural death is a cure for suffering	3,47	1,106	0,261	0,894	-0,506	-0,396

### Confirmatory factor analysis

**Fig. 1:**
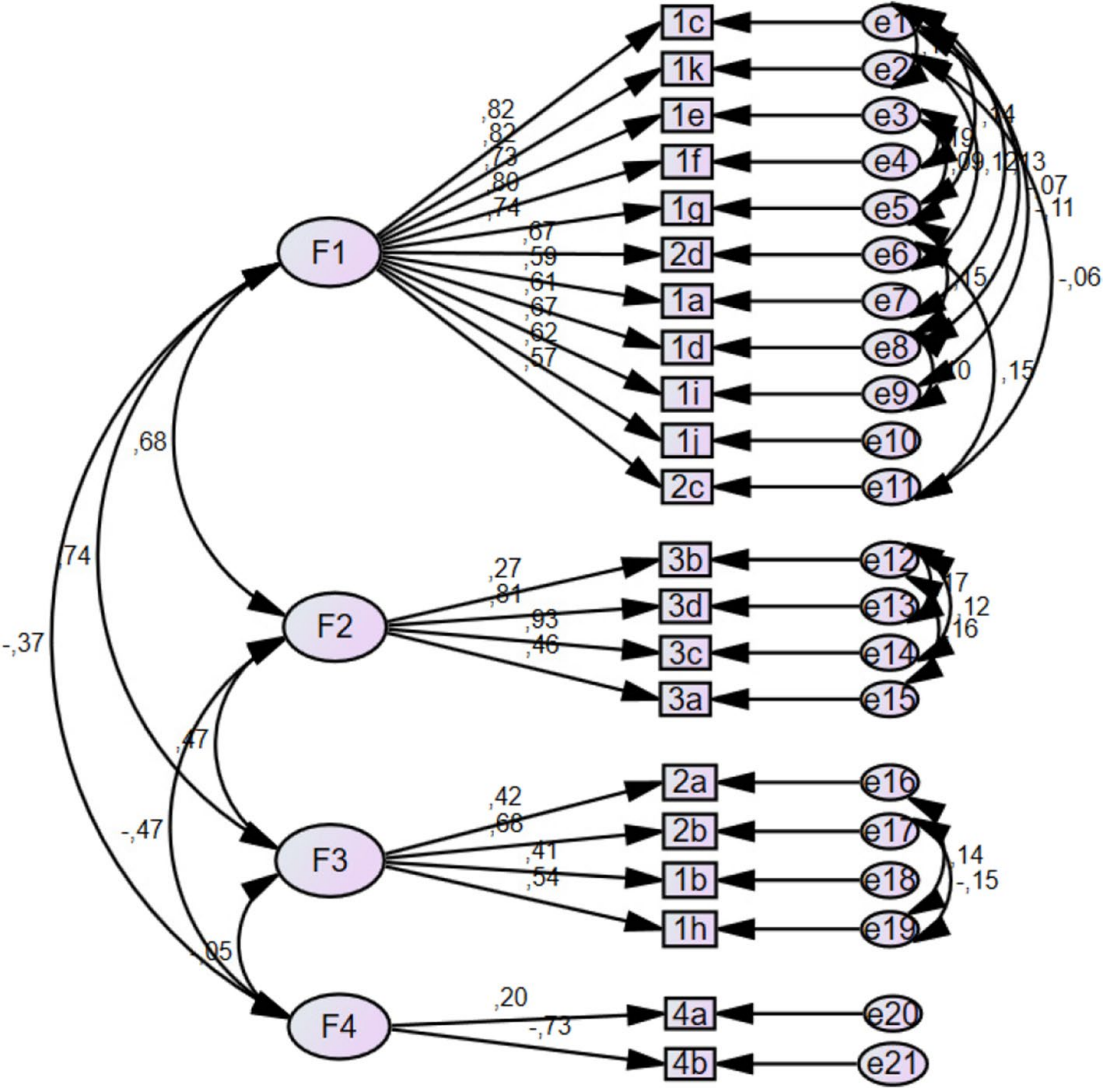
Four-factor structure of the EAS-ES-R questionnaire

A confirmatory factor analysis was carried out using principal components with varimax rotation, with a KMO of 0.938 and Bartlett's test of sphericity < 0.001, thus confirming the relevance of the analysis. Four components are considered, which can explain up to 56.99% of the variance (see Fig. 1).

For component 1, “ethical considerations” (items 1, 3, 4, 5, 6, 7, 7, 9, 10, 11, 14 and 15) explained up to 36.71%; for component 2, “appreciating life” (items 16 to 19) explained up to 8.5%; for component 3, “practical considerations” (items 2, 8, 12 and 13) explained up to 6.3%; for component 4, “naturist beliefs” (items 20 and 21) explained up to 6.3%; for component 3, “practical considerations” (items 2, 8, 12 and 13) explained 6.3%; and for component 4, “naturist beliefs” (items 20 and 21) explained 4.6% (see Fig. 1). The model shows an acceptable fit to the data, as reflected by several indicators, such as a CMIN/DF of 3.564. In addition, the RMSEA of the model was 0.056, with a 90% confidence interval ranging from 0.051 to 0.061 and a PCLOSE value of 0.025, suggesting that the model has a reasonable fit to the data from the discrepancy by degrees of freedom adjusted for sample size.

The analysis yields high NFI, RFI, IFI, TLI and CFI values (0.917, 0.897, 0.939, 0.924 and 0.938, respectively), confirming the model's fit to the data. On the other hand, measures such as the PRATIO, PNFI and PCFI showed values of 0.810, 0.742 and 0.760, respectively, indicating a good balance between goodness of fit and model complexity.

Table 5 shows the distribution of the items in the four components according to their loading factors.

**Table 5:** Loading factors of the questionnaire EAS-ES-R

	**1**	**2**	**3**	**4**
1.c	**0,807**	0,178	0,175	0,027
1.k	**0,786**	0,213	0,215	-0,055
1.e	**0,761**	0,104	0,159	0,106
1.f	**0,757**	0,192	0,228	0,033
1.g	**0,748**	0,191	0,108	0,170
2.d	**0,718**	0,163	0,092	-1,20
1.a	**0,661**	0,031	0,125	0,021
1.d	**0,549**	0,222	0,097	0,265
1.i	**0,548**	0,377	0,208	0,206
1.j	**0,545**	0,096	0,479	-0,137
2.c	**0,507**	0,454	0,032	0,011
3.b	-0,034	**0,717**	-0,038	-0,090
3.d	0,341	**0,679**	0,083	0,245
3.c	0,448	**0,640**	0,154	0,241
3.a	0,212	**0,592**	0,191	-0,063
2.a	0,086	-0,042	**0,781**	0,065
2.b	0,336	0,190	**0,552**	-0,105
1.b	0,67	0,330	**0,542**	-0,003
1.h	0,395	-0,106	**0,519**	-0,177
4.a	0,055	-0,107	0,044	**-0,839**
4.b	0,246	0,301	-0,134	**0,472**

### Parallel analysis

**Fig. 2:**
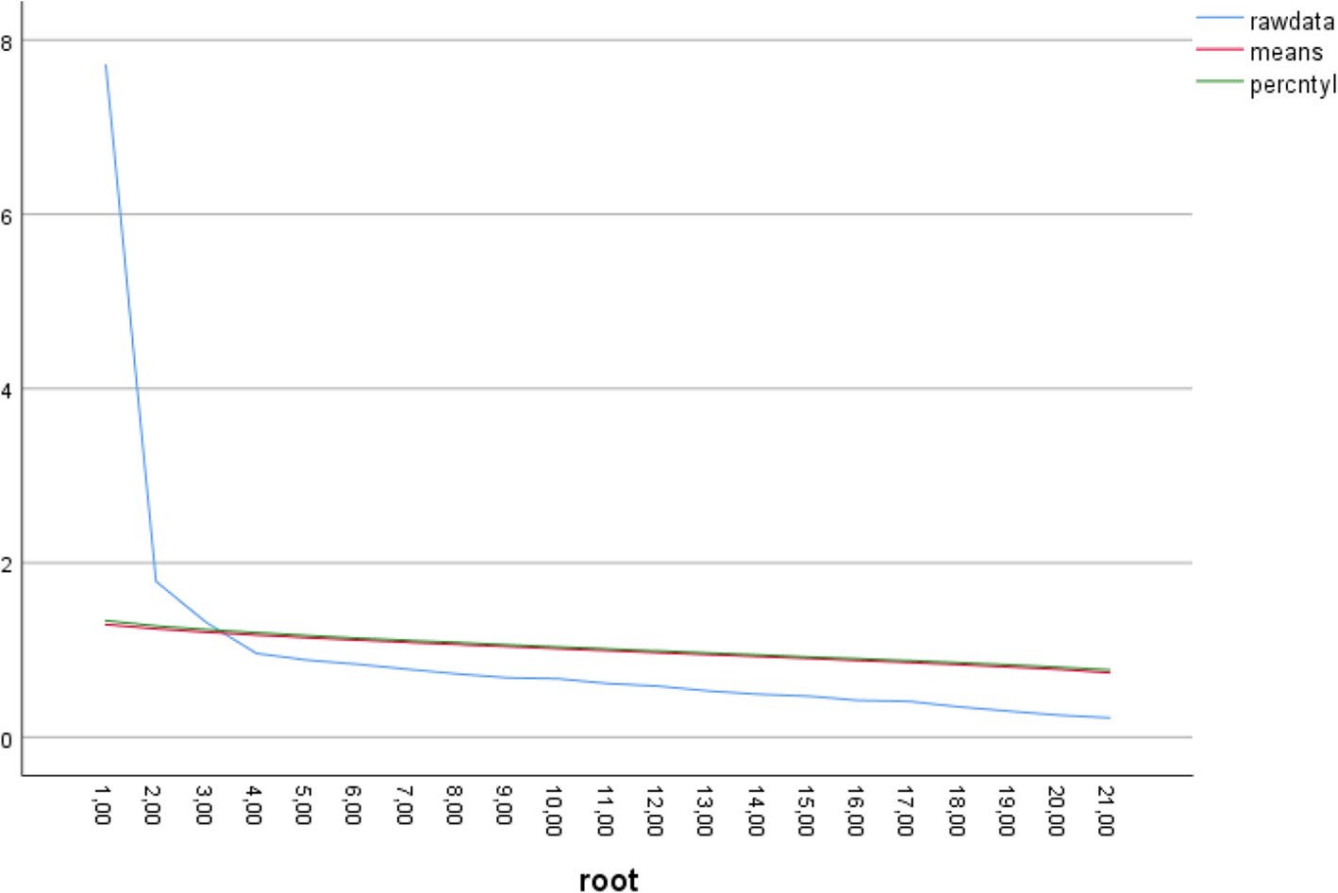
Parallel analysis

After parallel analysis was performed, three factors (those whose raw data were greater than the percentile value) were found. With 828 cases and 21 variables, 1000 parallel datasets were generated to establish a criterion for the number of factors to retain. The eigenvalues of the raw data show that the first three factors have significantly greater values (7.709925; 1.786161; 1.316861) than the randomly generated eigenvalues at the 95th percentile (1.337312; 1.275060; 1.236035), suggesting that the retention of these three factors is significant. From the fourth factor onward, the eigenvalues of the raw data approach or fall below the randomly generated eigenvalues, indicating that they may be less interpretive or significant (see Fig. 2).

## Discussion

The present study aimed to validate the questionnaire in Spanish after the partial modification of items 2, 4, 7 and 15. After the adaptation of the four new items, in the new EAS-ES-R version, we found a greater internal consistency (0.892 vs. 0.878) with respect to the EAS-ES version of Onieva-Zafra et al. [32]. In the first validation of the scale in Spanish, the sample consisted only of nursing students, whereas the EAS-ES-R has a sample that includes all health professionals of the Balearic Health Service and health professionals in training. On the other hand, this consistency result is in line with the findings of the original scale [30] revised by Rogers [31], where the value obtained for the Cronbach’s α was 0.84. Furthermore, we found reliability results in line with those of the instruments validated in Greece [33] (Cronbach’s α of 0.944), in Dutch [35] (Cronbach's α of 0.822), and in the Persian version [36] (Cronbach's α of 0.88), with the Chinese version [34] (Cronbach's α of 0.77) showing lower values for this parameter.

The resulting factor structure is similar to that of the EAS-ES version, with the migration of items 2c and 2d to form part of the first factor called “ethical considerations”, while items 1b and 1 h are removed from this factor. For items 2c and 2d, this can be explained by the fact that the context in reference to the regulation of euthanasia has changed, as it is already regulated in Spain. Although the translation and validation processes in the different languages yielded disparate results in terms of the number of factor loadings and the inclusion/exclusion of items, we can see how the revision of the original Rogers questionnaire [31] yielded 9 items that met the criteria for interpreting the questionnaire. The first factor obtained, "general moral and legal acceptance", had 4 of its 5 items within the "ethical considerations" factor of our questionnaire. The missing item "God gave us life and should be the only one to end it" was not included in the EAS-ES version. As reflected in the validation of the EAS by Aghababaei [36], religious arguments are shown to be the most powerful reasons for opposition to euthanasia [37–40]. Items 1b and 1 h move to factor 3, called "practical considerations", which is also marked by the context of the regulation of euthanasia in Spain, leaving their ethical imprint and acquiring a greater practical sense. Factor 1b is also one of the items modified in the new Spanish version and loses the religious charge of the expression "merciful reason" for "compassion", which is a more universal value and is not as affected by religious thought. The rest of the items remained grouped in the same way as in the first Spanish version in terms of the factors "appreciating life" and "naturalistic beliefs". The variations in the concepts explained have helped to improve the internal validity and reliability of the instrument and better adapt it to the new regulatory context in reference to the provision of aid in dying in Spain.

Regarding the number of loading factors, we found that the total of four obtained values are consistent with the previous Spanish version and with the work of Chong et al. [34] and Demedts et al. [35]. However, the Greek version [33] has 5 loading factors, and the revision of the original English version [31] and the Persian version [36] has 3 factors. These differences may be marked by the total number of respondents, the type of sample and the fact that some items show great variability in their explanatory power. If we look at previous validations of the instrument, we can observe that the studies with the highest number of respondents (32,34,35) are those with 4 factor loadings. However, although they present 4 factors, it is difficult to establish an exact parallelism in their configurations. This is especially visible in Chong's study (34), where despite finding 4 factors, 13 new items were introduced, for a total of 31. Furthermore, the factors have been named "General Euthanasia", "Passive Euthanasia", "Active Euthanasia" and "Non-Voluntary Euthanasia", terms that do not correspond to the bioethical conceptual content of the current debate in reference to the MAID. At present, the concepts "Passive Euthanasia" and "Active Euthanasia" are not used in bioethical language, referring only to the concept of Euthanasia or assisted suicide.

If we take into account that beliefs and attitudes about ethical dilemmas related to the end of life are not static and may vary throughout an individual's life, we can understand that the sociocultural context, undergraduate and postgraduate professional training and other sociodemographic determinants may significantly shape attitudes toward euthanasia among health professionals. Thus, the instrument shows its sensitivity to these changes in attitudes toward euthanasia in a particular context and shows how items may migrate from one factor to another because euthanasia is regulated in that particular context.

As is also shown in the bioethics literature, "religiosity" is also a factor impacting attitudes toward euthanasia [37–41]. In the present study, we found that nonreligious people showed more favorable attitudes toward euthanasia. This is also reflected in other studies, such as that of Terkamo-Moisio et al. [27] in 2019, in which postgraduate nurses were surveyed and found that being considered religious had a negative impact on attitudes toward their caring role in the euthanasia process, which meant that they were less well prepared for the provision of care in this context. Arreciado et al. [42], in 2024, also related in their study the fact that they did not consider themselves religious to better attitudes toward euthanasia among nursing students. This predictive value of the variable "being religious" for showing different types of attitudes toward euthanasia has also been demonstrated in other previous work [43–45].

Although Law 3/2022 on the Regulation of Euthania in Spain defines the context in which this practice can be carried out, it is true that, as Rogers [31] pointed out, the questionnaire instructions do not provide a definition of the concept of "euthanasia", which could guide the participants in the study. The fact that 68.3% of respondents acknowledged knowing little or nothing about this concept could indicate that if certain respondents were clear about this concept, they might have more favorable attitudes toward euthanasia within the health system. For this reason, it seems to be very important to develop informative and educational campaigns from the National Health System as well as in the centers where health professionals are trained.

## Conclusion

The revised EAS-ES resulting from this study is a valid instrument for assessing attitudes toward euthanasia, with psychometric characteristics similar to those reported in the international literature. The EAS-ES-R can be used in different contexts for Spanish-speaking healthcare professionals, as it has shown significant internal consistency in a sample composed of different healthcare professions and students of healthcare professions or those studying a specialty. This makes it an important tool for detecting different attitudes toward the euthanasia and therefore for supporting the professionals who form part of the teams that provide services in the process of aid in dying and health management in the development and implementation of Law 3/2022 on the regulation of euthanasia.

### Limitations

This study has some limitations to take into account. Initially, it was important to recognize that the EAS is a self-report instrument that might be less accurate in assessing opinions as participants may not respond truthfully in a way to present themselves in a socially acceptable manner. Another limitation is that the questionnaire have not provide the participants with a definition of euthanasia. Finally, although the sample is predominantly composed of nurses, it is not representative of all healthcare profession sectors.

## Data Availability

The datasets generated and analysed during the current study are not publicly available due privacy and ethical restrictions of the participants, but are available from the corresponding author on reasonable request.
